# Supplementary figures and images for: Multidimensional analysis and detection of informative features in human brain white matter
Source: PLoS Comput Biol. 2021 Jun 28;17(6):e1009136. doi: 10.1371/journal.pcbi.1009136 (PMC8270416; doi:10.1371/journal.pcbi.1009136)

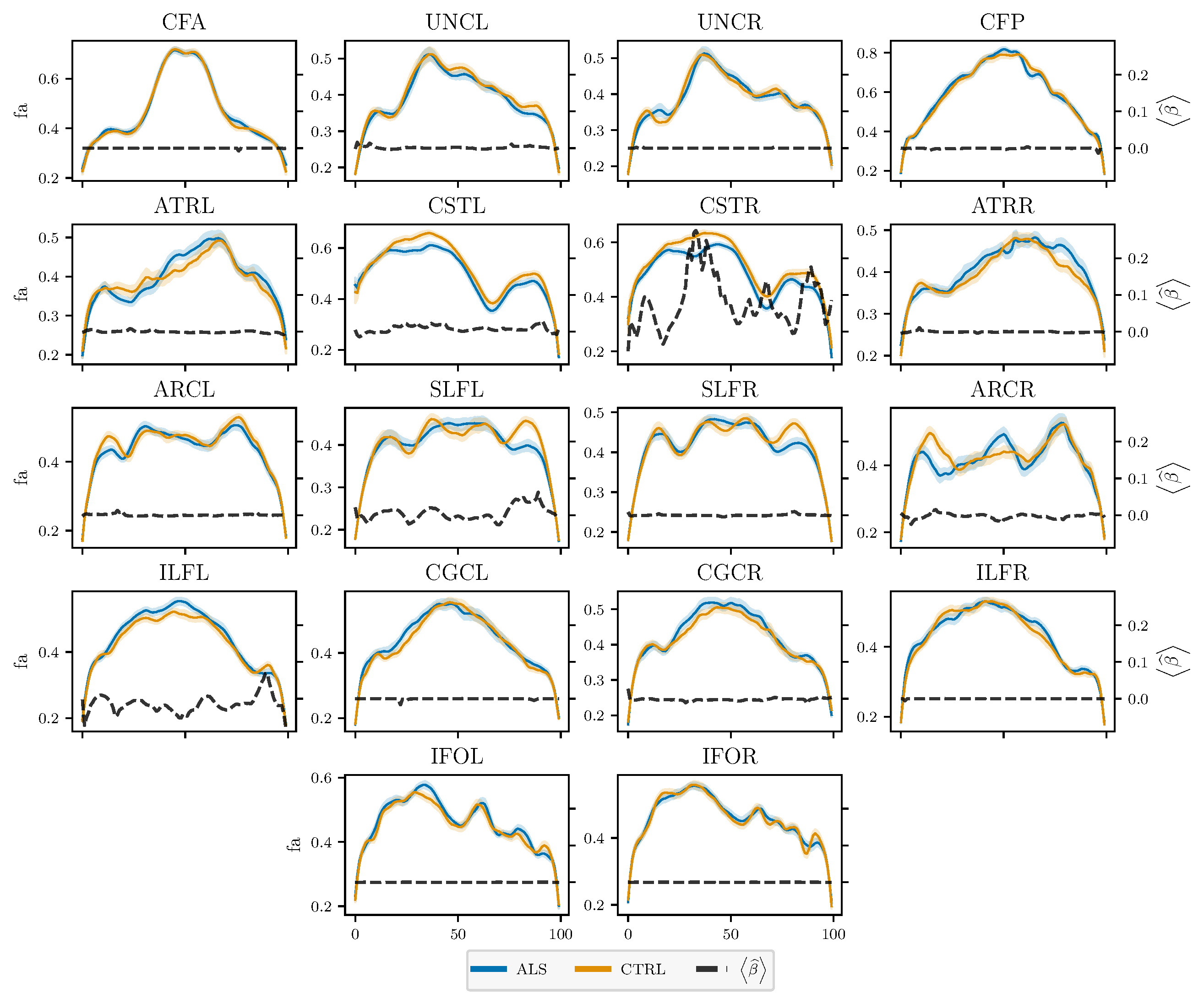

Supplement: S1 Fig — Diffusion metrics are plotted along the length of eighteen bundles: right corticospinal (CSTR), left corticospinal (CSTL), right uncinate (UNCR), left uncinate (UNCL), left inferior fronto-occipital fasciculus (IFOL), right inferior fronto-occipital fasciculus (IFOR), right arcuate (ARCR), left arcuate (ARCL), right thalamic radiation (ATRR), left thalamic radiation (ATRL), right cingulum cingulate (CGCR), left cingulum cingulate (CGCL), callosum forceps posterior (CFP), callosum forceps anterior (CFA), right inferior longitudinal fasciculus (ILFR), left inferior longitudinal fasciculus (ILFL), right superior longitudinal fasciculus (SLFR), and left superior longitudinal fasciculus (SLFL). FA is plotted on the left y-axis while the β^ coefficients are displayed on the twin axis on the right-hand-side. SGL selected the right corticospinal tract (CSTR) as important and regularized coefficients in the CSTL. Yet, there are also group FA differences in the CSTL. This highlighted a potential drawback of the SGL method, discussed in the main text in the context of age regression. Namely, SGL is not guaranteed to identify all important features. In this case, if the diagnostic signal in the CSTL is redundant to that in the CSTR, SGL will regularize the CSTL features, thereby reducing its sparsity penalty without any corresponding increase in loss. This parsimony cuts both ways; it is a feature of the method when one seeks an efficient predictive model, but is a disadvantage of the method when one wants an exhaustive explanation of feature importance. We use the phrase “parsimony pitfall” to refer to the case when SGL regularizes away redundant but obviously important features. (TIF) [file pcbi.1009136.s001.tif]

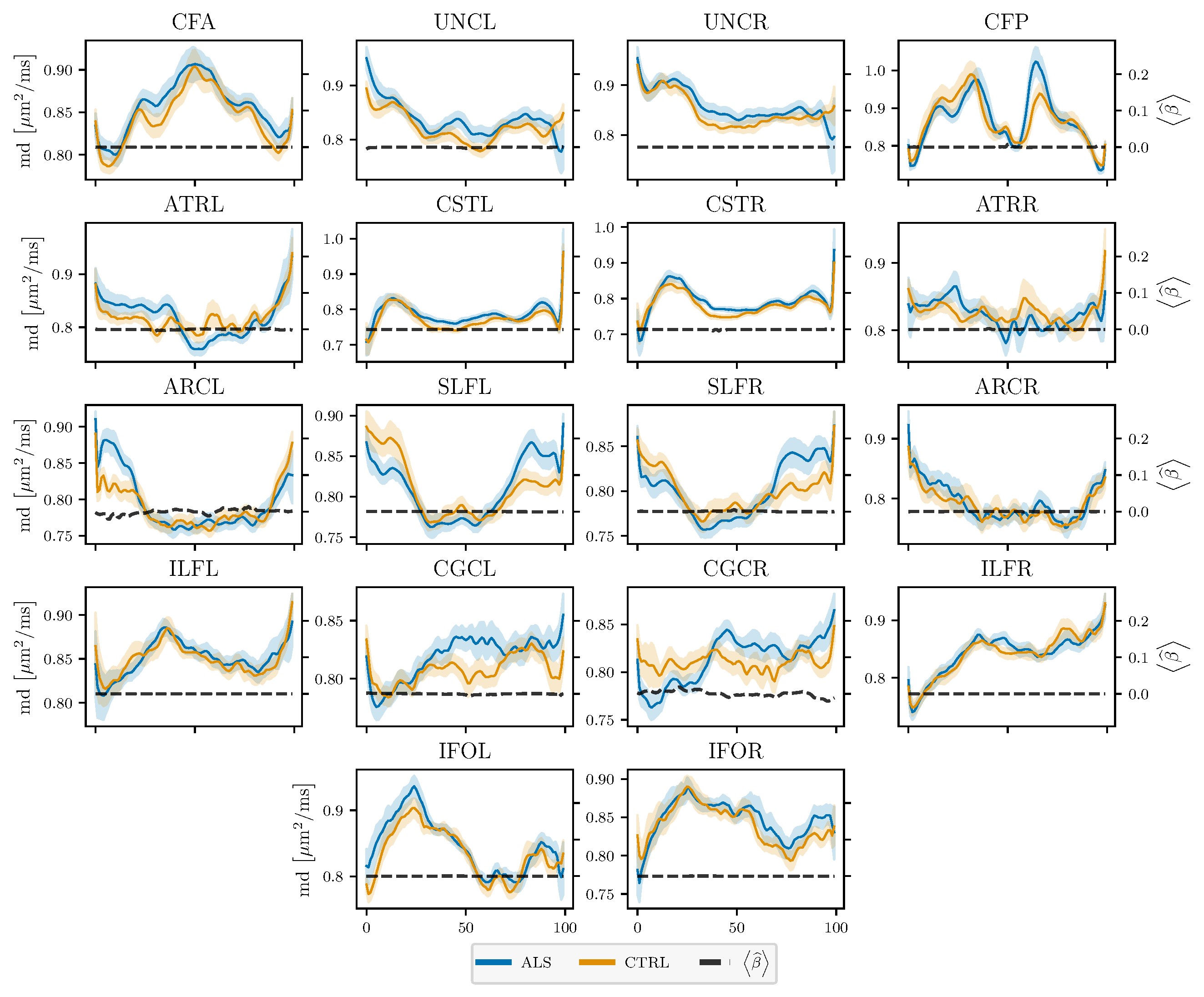

Supplement: S2 Fig — The scale of the β^-axis is identical to that used in S1 Fig, to facilitate the comparison of the relative importance of each metric. (TIF) [file pcbi.1009136.s002.tif]

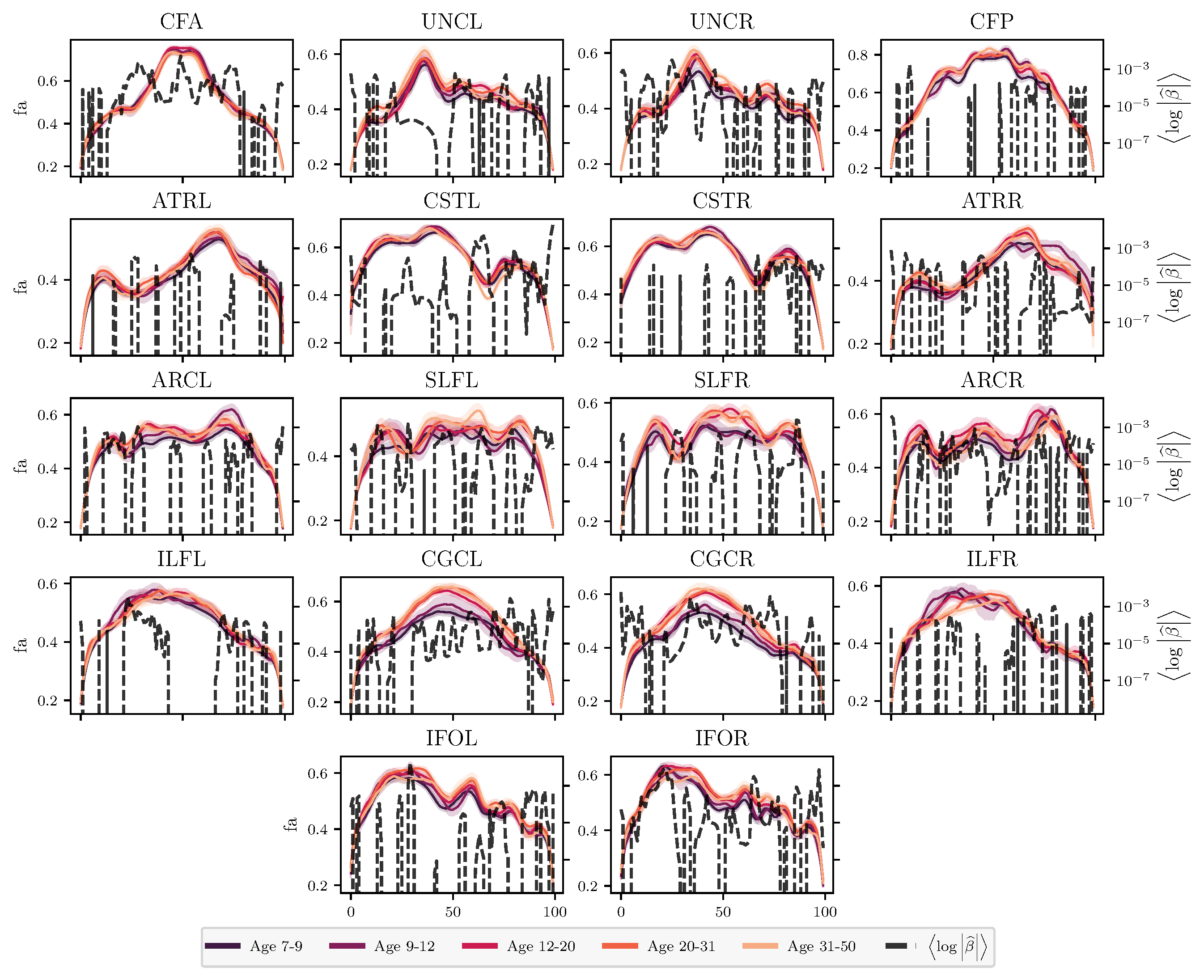

Supplement: S3 Fig — In contrast to the ALS classification case, the β^ coefficients are distributed widely through the brain, supporting the interpretation that aging is a large and continuous whole-brain process. (TIF) [file pcbi.1009136.s003.tif]

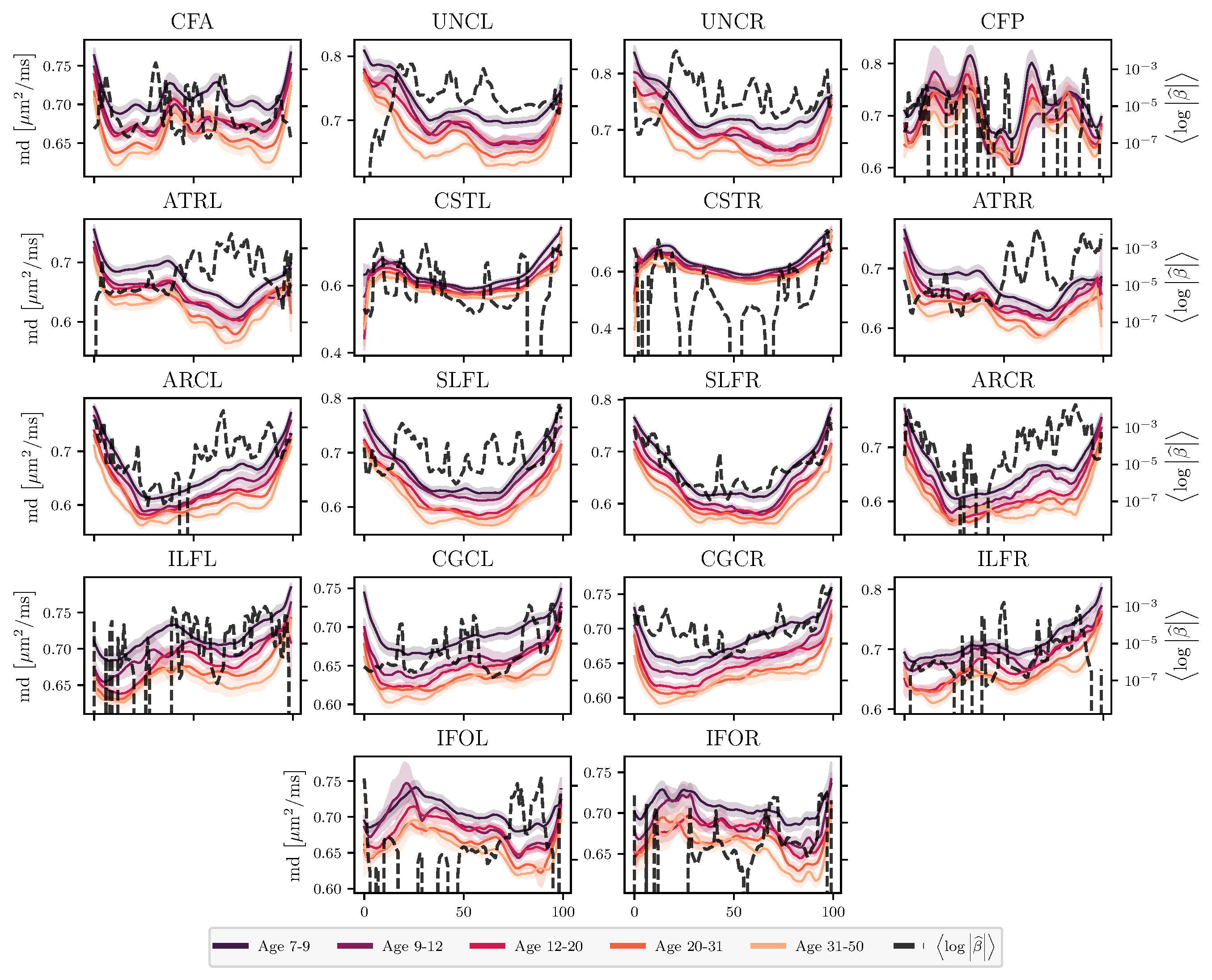

Supplement: S4 Fig — This and S3 Fig demonstrate that SGL behaves much more like the lasso than the group lasso, as discussed in the main text. The parsimony pitfall is most evident in the IFOL and IFOR bundles. (TIF) [file pcbi.1009136.s004.tif]

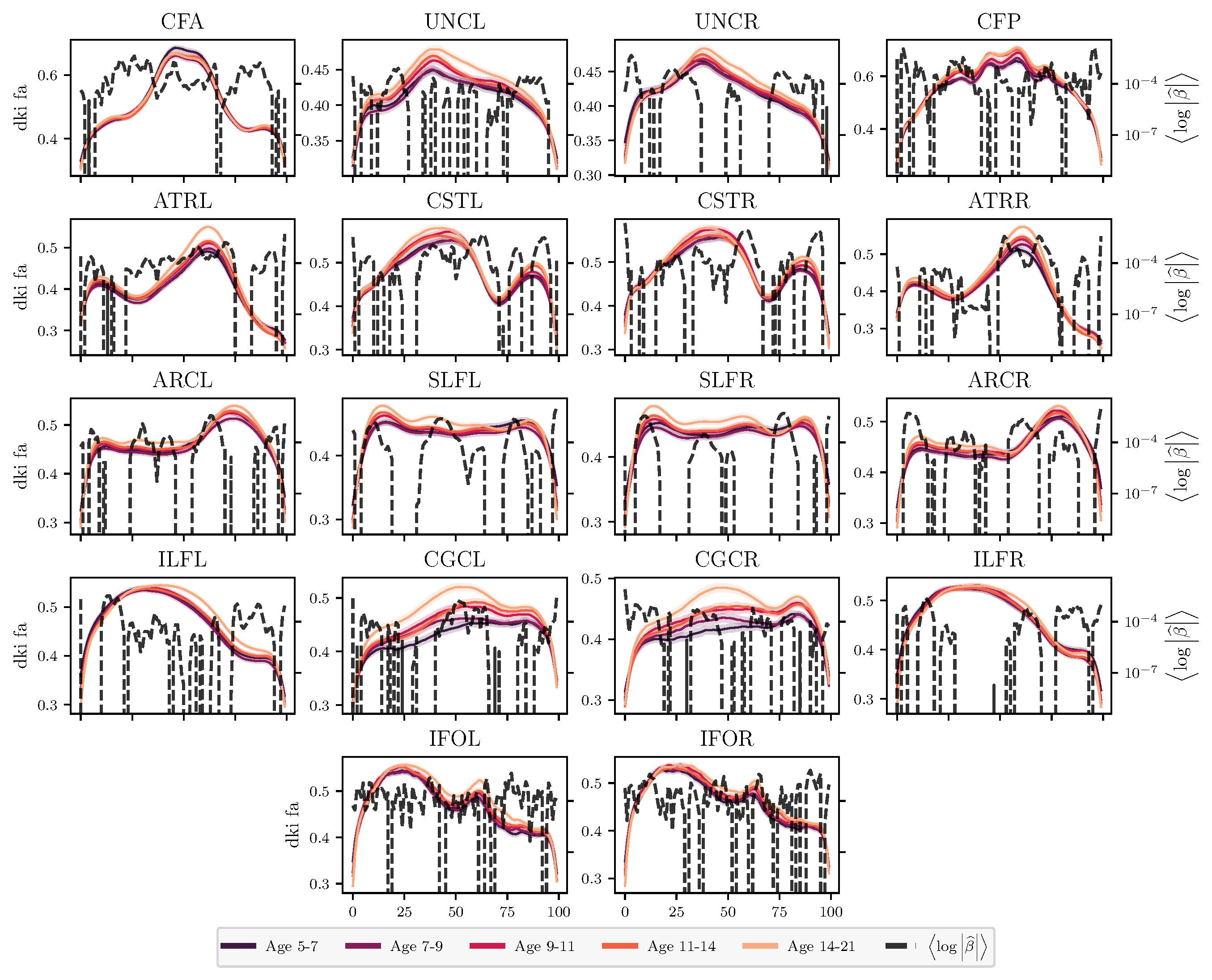

Supplement: S5 Fig — Like the WH dataset, the β^ coefficients are distributed widely through the brain and SGL behaves more like the lasso than the group lasso. (TIF) [file pcbi.1009136.s005.tif]

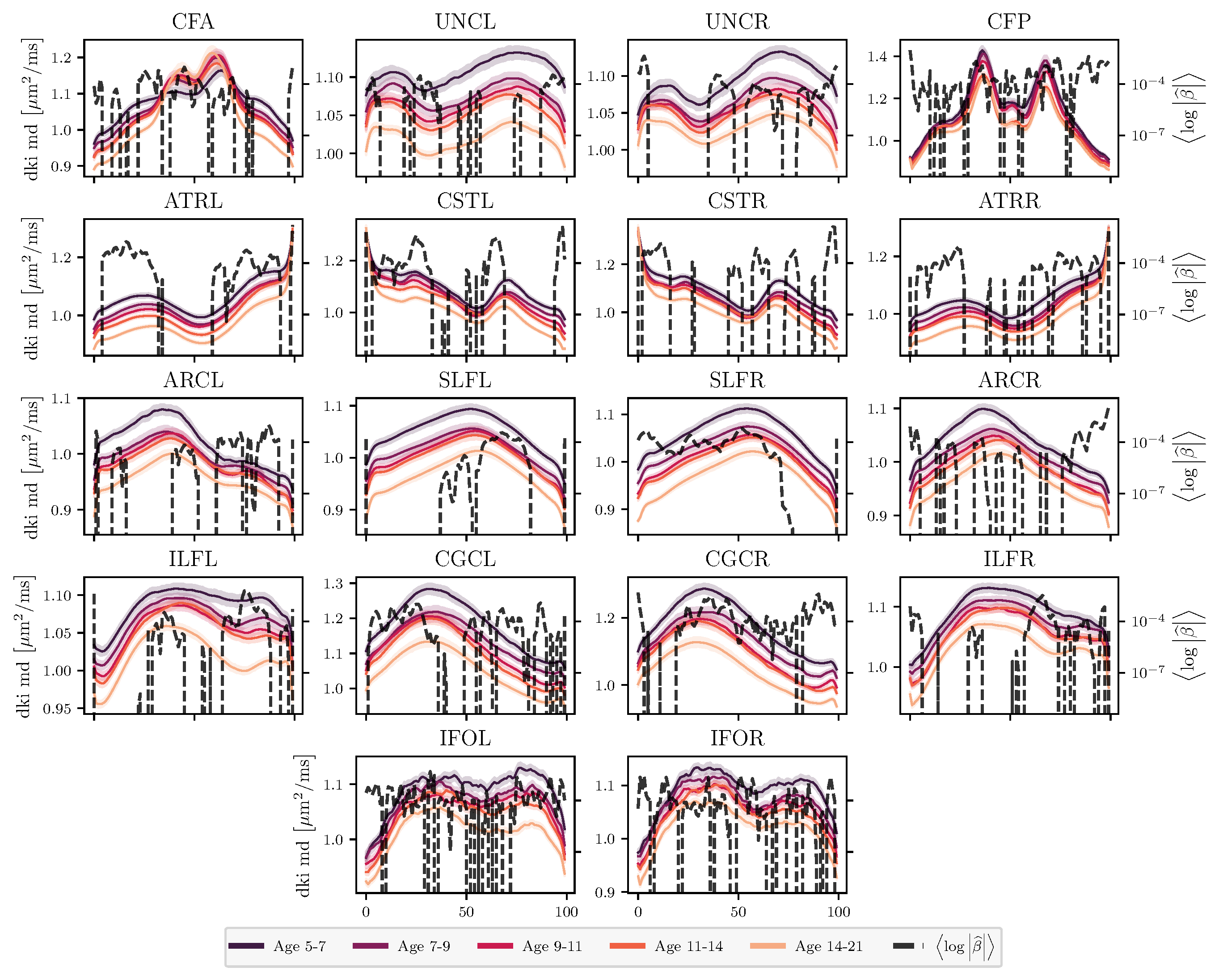

Supplement: S6 Fig — In contrast to the WH results, the bundle profiles show different behaviors. For example the SLFL and SLFR bundle profiles in have different concavity. This is unsurprising, however, given the differences between these datasets (see also Discussion). The parsimony pitfall is most evident in the UNCL, UNCR, ARCL, SLFL, and SLFR bundles. (TIF) [file pcbi.1009136.s006.tif]

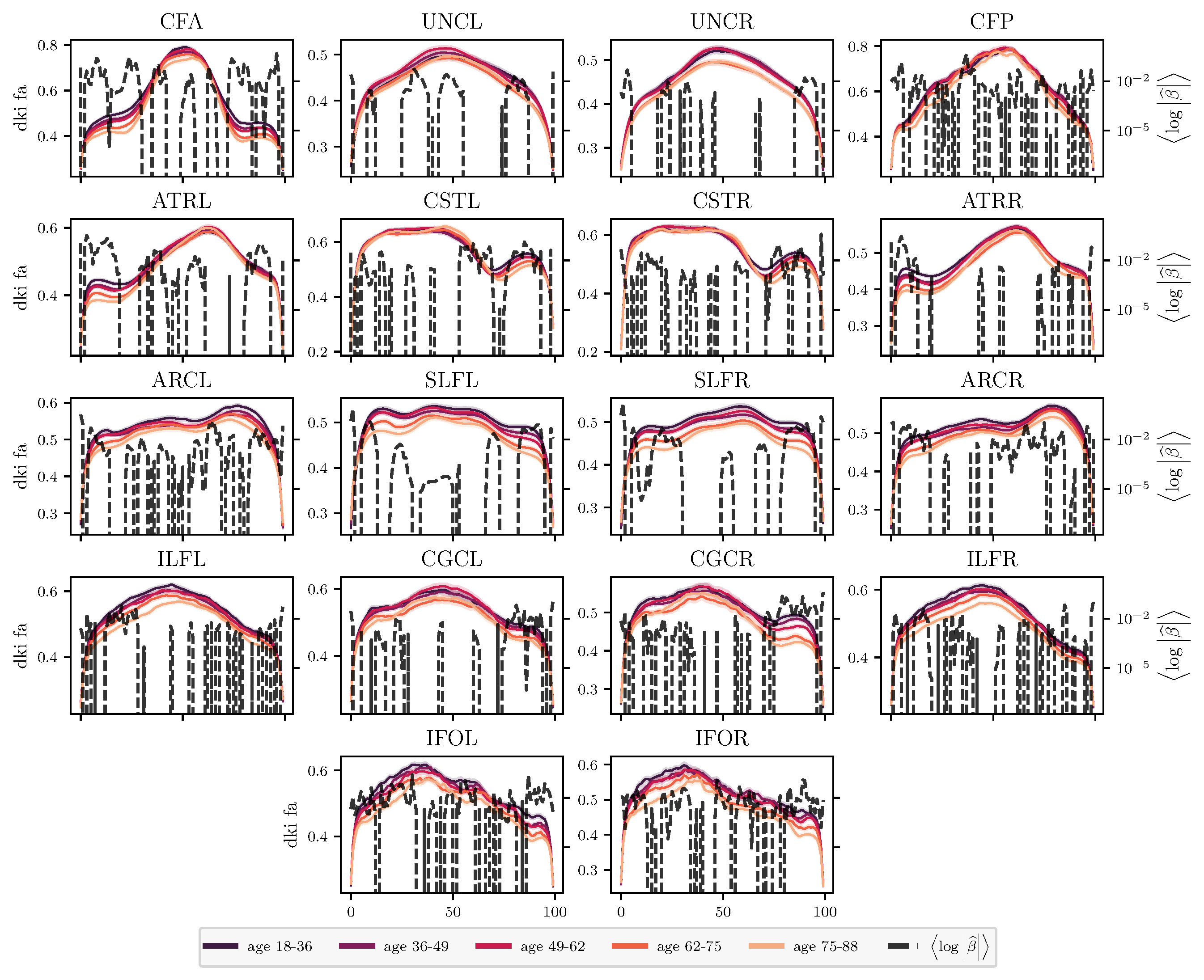

Supplement: S7 Fig — The β^ coefficients are distributed widely through the brain and SGL behaves more like the lasso than the group lasso. As before, one must be cautious about comparing bundle profiles and β^ coefficients between models. While the HBN and Cam-CAN datasets share the same diffusion model and refrain from clipping streamlines, the age distributions for the two are roughly disjoint, with the WH age distribution straddling the two. (TIF) [file pcbi.1009136.s007.tif]

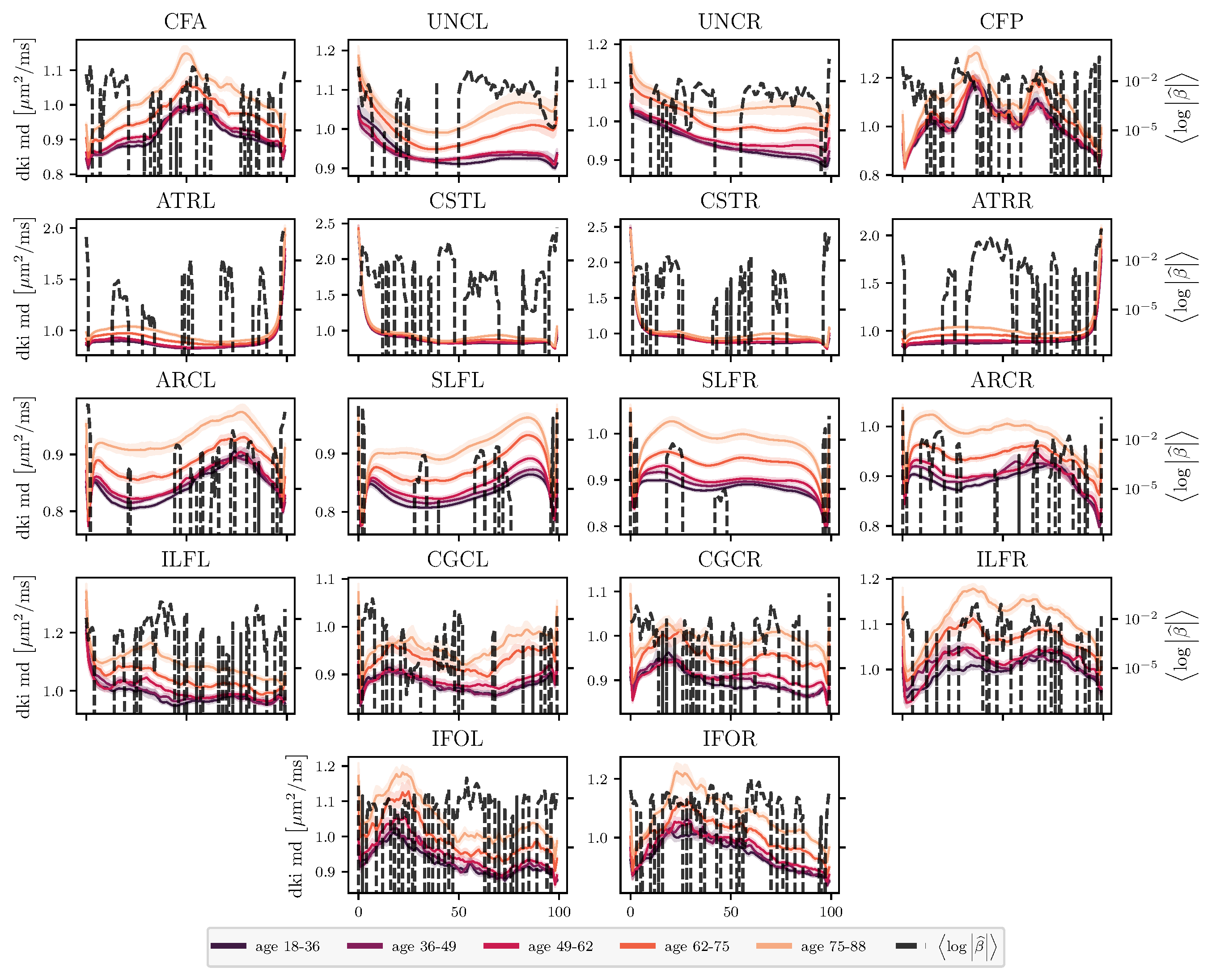

Supplement: S8 Fig — (TIF) [file pcbi.1009136.s008.tif]

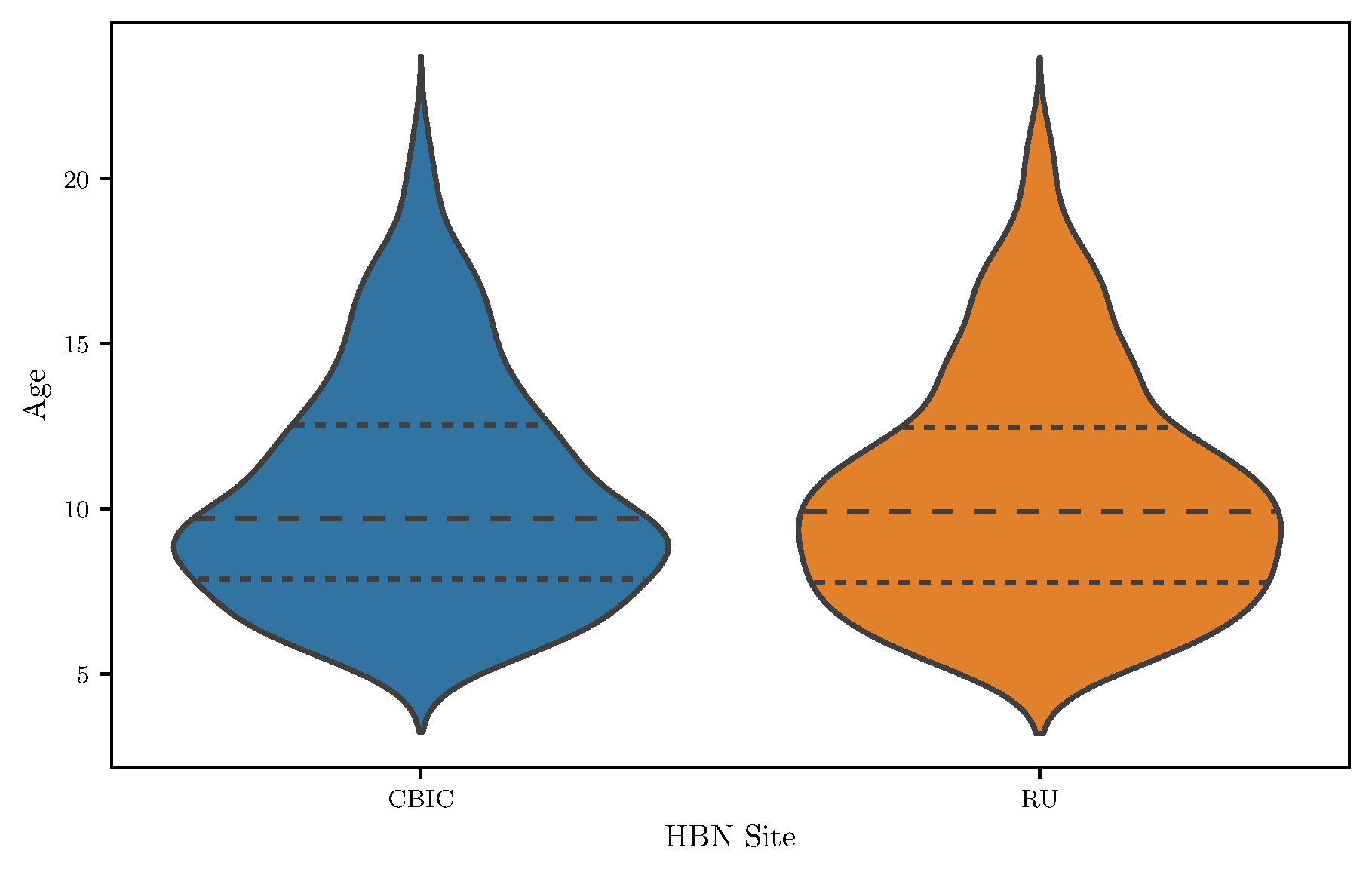

Supplement: S9 Fig — Rutgers Rutgers University Brain Imaging Center (RU) and the CitiGroup Cornell Brain Imaging Center (CBIC). (TIF) [file pcbi.1009136.s009.tif]

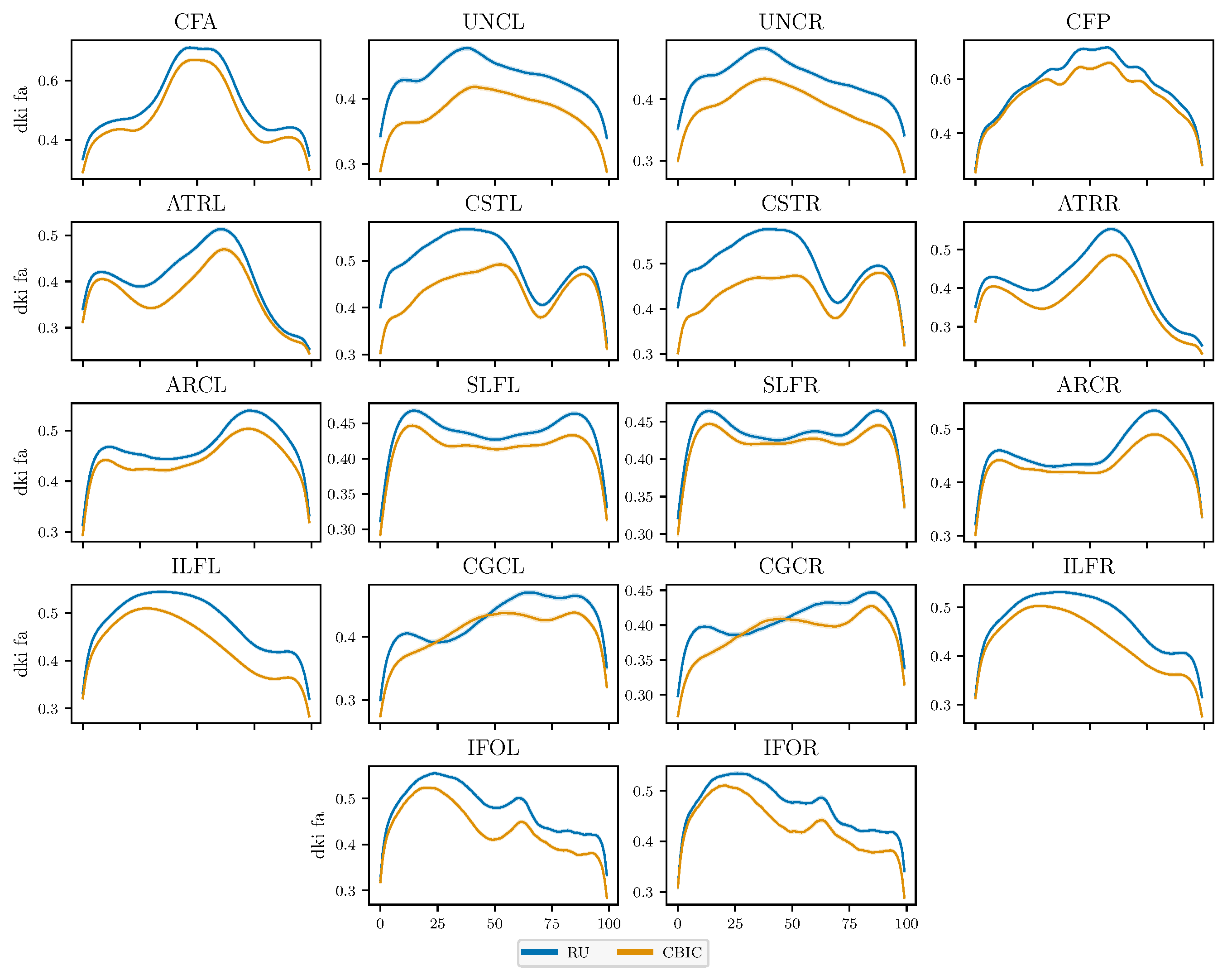

Supplement: S10 Fig — (TIF) [file pcbi.1009136.s010.tif]

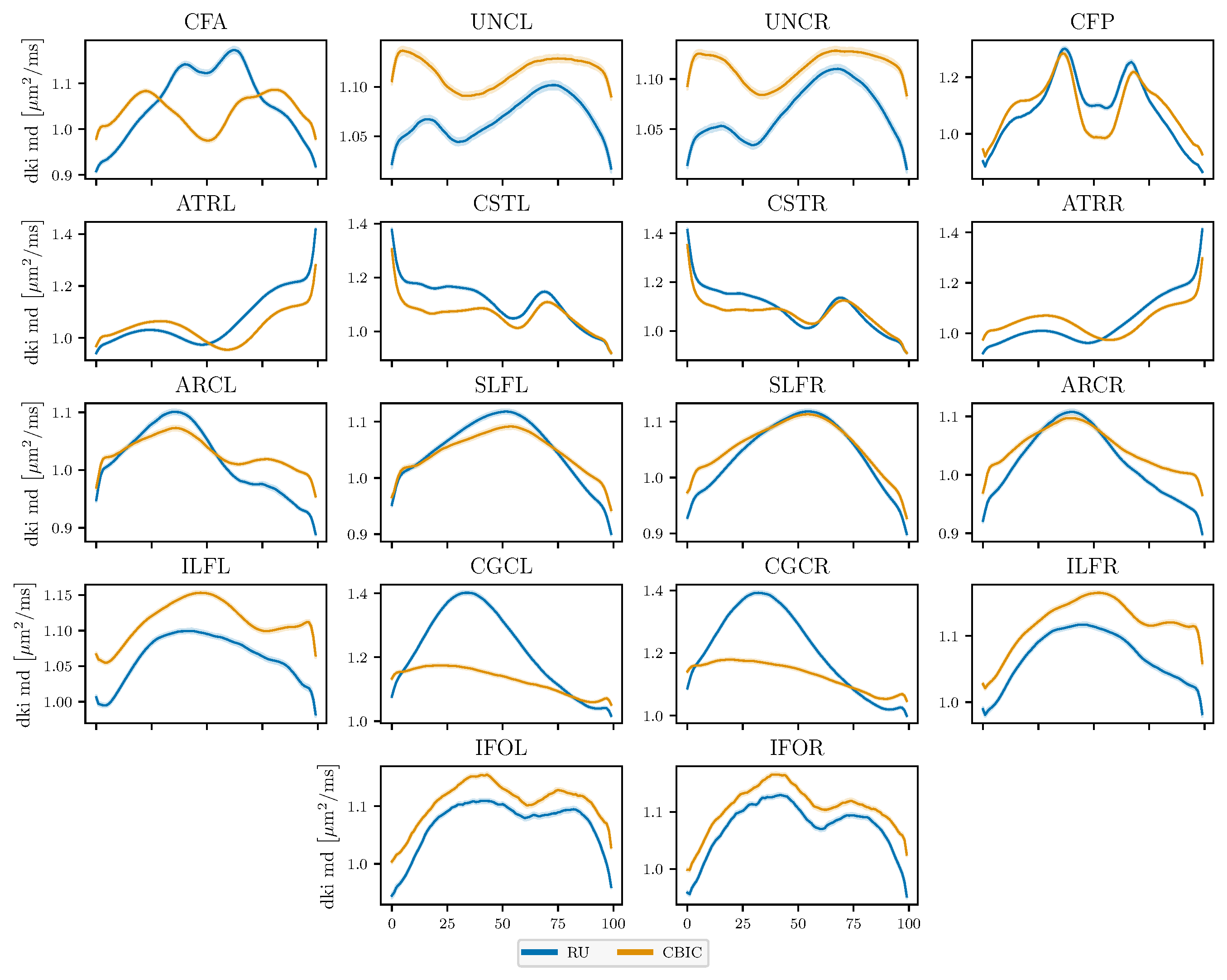

Supplement: S11 Fig — (TIF) [file pcbi.1009136.s011.tif]

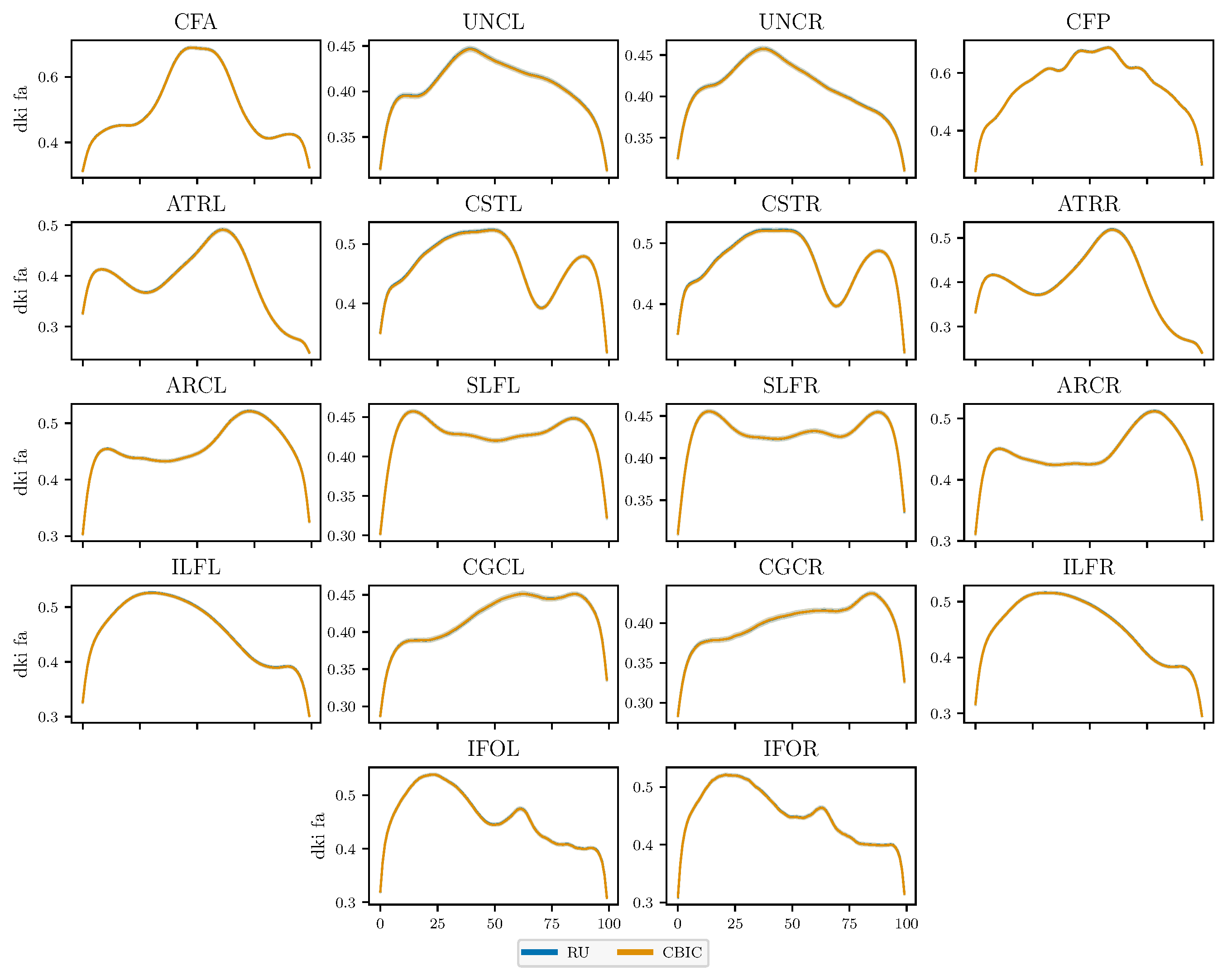

Supplement: S12 Fig — (TIF) [file pcbi.1009136.s012.tif]

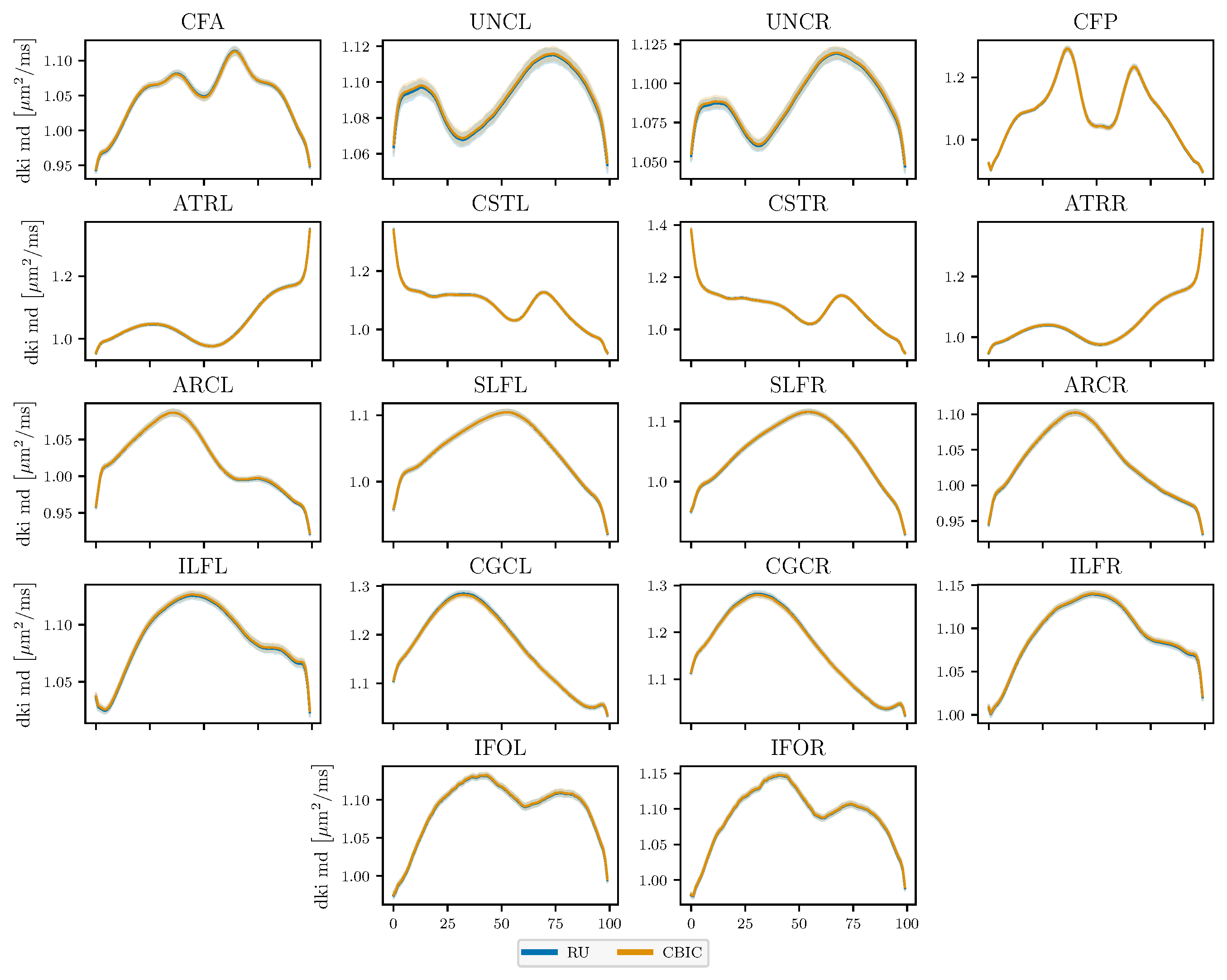

Supplement: S13 Fig — (TIF) [file pcbi.1009136.s013.tif]
