# Supplementary material for: Multidimensional analysis and detection of informative features in human brain white matter
Source: PLoS Comput Biol. 2021 Jun 28;17(6):e1009136. doi: 10.1371/journal.pcbi.1009136 (PMC8270416; doi:10.1371/journal.pcbi.1009136)
Supplement: Attachment — Submitted filename: R1ReviewerComments.pdf [file pcbi.1009136.s014.pdf]

We appreciate the comments made by the reviewers and the editors and we thank them for the time they took to read our work and to provide constructive feedback. We were pleased to see that all three reviewers found our work valuable. For example, reviewer #1 said:

“Overall, I found the results to be quite compelling”

Reviewer #2 commented that:

“Overall, the framework provides a great addition to the existing AFQ software suite, and I find it worthy of publication at PLOS Computational Biology...”

And reviewer #3 noted:

“The method is interesting and very sound and can be very useful for neuroscientists. The manuscript is well written and explains quite clearly the method, experiments, and results.”

In the following, we include a point-by-point response to the reviewers’ comments, including all of the actions that we have taken to address these comments. Reviewer comments remain in black typeface, while our responses are added in blue typeface. We have also attached the revised manuscript, both with and without changes highlighted. The figures have been removed from the pdf file and attached separately to comply with PLOS Comp Bio figure requirements.

Point-by-point response

Dear Authors,

Thank you for submitting your article. Three reviewers have provided their comments and they are all positive about the quality of your contribution. They ask, however, for some additional precision in the discussion of alternative methods and the description of the results that I encourage you to take into consideration, as they should help increase the impact of your work. We will be looking forward to receiving a revised version of your manuscript, taking these comments into account.

Best regards,

Roberto Toro

Thank you for this assessment. In response to reviewer comments and suggestions, we have substantially revised the manuscript. Here, we summarize the major changes that we have made and provide further details in response to reviewer comments below:

1. Following comments from reviewer #1 and reviewer #3, we added four new statistical analysis methods: Lasso PCR, Elastic Net, Bundle mean Lasso and the newly-derived PCR-SGL. We provide an evaluation and comparison of the different methods.
2. We elaborated on the distinction between inference and prediction (or “decoding” and “encoding”) in the Introduction in response to reviewer #1’s comments.
3. We modified the figures for clarity in response to comments from all three reviewers.
4. We reran the analysis on the HBN dataset, using ComBat harmonization, partially addressing concerns raised by reviewer #1. We also added Discussion of cross-dataset incongruencies in response to these concerns.
5. We extended the list of limitations in the discussion section in response to reviewer #3.

Reviewer #1:

This article describes a systematic evaluation of sparse group lasso (SGL) applied to tractometry output of dMRI-derived streamlines as a classification and individual differences method. Using SGL+tractometry in the classification setting, the authors reliably distinguish between ALS patients and matched controls at a classification accuracy of 83%, with the major contributing pathways to classification being the descending corticospinal (CST) pathways, as would be expected for an upper motor neuron disease. In the regression context, the authors found that they could reliably predict chronological age in 3 different data sets, with r^2’s ranging from 0.52-0.77. The authors conclude that this approach provides an effective way of identifying white matter phenotypes from dMRI data that also identifies contributing pathways relevant to model accuracy.

Overall, I found the results to be quite compelling. There are some outstanding questions about how this method relates to prior approaches and details about the modeling work itself that should be addressed. But in general I think this work will be a valuable contribution to the field.

MAJOR COMMENTS

- The authors make a compelling case that standard voxelwise approaches to developing brain-based phenotypes, particularly from dMRI data, are quite limited due to their high dimensionality and lack of specificity. One thing that is not clear up front, and could help in the interpretation, is that the authors are specifically talking about it in the context of decoding models (e.g., Y = group/individual measure), unlike many previous models (e.g., TBSS, connectometry) that are encoding models (e.g., Y= brain measure). For example, when I first read the Introduction, the discussion of prior approaches not characterizing specificity of bundles relevant to individual differences seemed incorrect as TBSS, connectometry, and even the initial tractometry approaches do this to some degree (and even control for the multiple comparisons). But the authors are correct that there are not many approaches that try to answer

decoding questions while maintaining inferential specificity of individual pathways. Since the field is largely dominated by encoding models, I recommend making this clearer up front to avoid similar confusion in the future.

DISCUSSION: We agree that this is an important distinction, but rather than use the terms “decoding” and “encoding” that are, if we understand correctly, based on the functional MRI literature, we would like to couch this distinction in the terms “inference” and “prediction” as used in Bzdok, Engemann and Thirion’s “Inference and Prediction Diverge in Biomedicine” (Patterns 1, 100119, November 13, 2020).

ACTION: We added a paragraph (starting on line 52) discussing this distinction in the Introduction, using Bzdok et al. as a reference. We also refer to a review by Naselaris et al. (2011) to make the connection to “encoding” and “decoding” models in fMRI explicit.

- Along the same lines as my prior comment, I wonder if the authors could compare their approach to the commonly used sparsity-constrained PCR (i.e., LASSO PCR) approach. This is becoming widely used in neuroimaging and has been applied specifically to dMRI data (see: 2) Powell, M. A., Garcia, J. O., Yeh, F. C., Vettel, J. M., & Verstynen, T. (2018). Local connectome phenotypes predict social, health, and cognitive factors. Network neuroscience, 2(1), 86-105. 2) Rasero, Javier, Amy Isabella Sentis, Fang-Cheng Yeh, and Timothy Verstynen. "Integrating across neuroimaging modalities boosts prediction accuracy of cognitive ability." PLOS Computational Biology 17, no. 3 (2021): e1008347.)*. I do not mean to vet directly against the SGL+tractometry method itself, but maybe add a section in the Discussion describing how this method compares to other common multivariate approaches used in computational neuroimaging like LASSO-PCR.

*Full disclosure: I am the senior author of these papers and am fully aware of how annoying it is for reviewers to ask that you cite their work. I’m not asking for citations per se, but a comparison of these PCR-based methods.

DISCUSSION: Thank you for the suggestion! First off, we have added those two PCR Lasso citations to the text. Reviewer #1 explicitly said he was not asking for citations but his suggestions were made in good faith and the citations were highly relevant to the work we did in response to the comment so they belong in the paper.

We view the Lasso PCR as somewhat orthogonal to and perhaps complementary to group sparse methods. Our SGL approach seeks to establish whether prior knowledge of anatomical grouping improves the modeling of brain-phenotype relationships. Conversely, the Lasso PCR approach seeks to model brain-phenotype relationships using the most parsimonious representation of variance in the diffusion measures. We ran a Lasso PCR model on our four datasets. For the ALS classification case, it performed poorly compared to all other models. This makes sense, since the ALS signal is mostly confined to a single bundle and the principal components are likely capturing whole brain variance that are not related to the disease.

Conversely, for age regression Lasso PCR performed as well as or better than SGL, depending on the dataset.

This motivated us to evaluate whether the PCR approach could be implemented while retaining the anatomical grouping information. We call this approach group-PCR SGL. Within each group (i.e. bundle metric combination), we perform PCA, keeping the maximum number of components. Because the group-wise PCA retains the group structure of the original data, we can still use SGL.

ACTION: We ran Lasso PCR and added it to the list of comparison models. We also created PCR-SGL models to evaluate whether we could adopt the PCR approach while also retaining group sparsity constraints. We discuss the advantages and limitations of these methods in the Discussion. These changes start at line 85 in the Introduction, 112, 129, 203, and 210 in the Results, the new Figure 4, line 255 in the discussion, and line 524 in the Methods.

- The comparison of the SGL approach to traditional lasso seems limited. Traditional lasso will pick the best predictor out of a correlated set of predictors. Adding in the group constraint allows for keeping some degree of correlations/clustering in the final model (a good assumption when building predictors from brain imaging data sets). It seems to me that the appropriate comparison would be against a ridge model, since ridge allows for clustering of predictors, but in a different way than group lasso does. If I were to place money, my guess is that the performance of a ridge or elastic net model would sit somewhere in the middle of the lasso results and SGL results.

DISCUSSION: This is a good comparison to make since SGL's convex combination of the lasso and group lasso penalties lead to an elastic net fit within each non-zero group (see Simon et al. 2013). In response we added the elastic net to our list of comparison models. With enough training data reviewer #1's bet is spot on, the elastic net models perform somewhere in between the pure lasso and SGL results. For the WH and ALS datasets with limited training data, the elastic net models perform about as well as the pure lasso models.

ACTION: We added elastic net to the list of comparison models and discussed the comparison in the results section. This change is in lines 129 and 203 in the Results as well as in the new Figure 4.

- There is substantial variability in the contributing pathways for age prediction across data sets. This seems incredibly important, but overlooked. One of the interpretive validations of the classifier results is that the pathways contributing to the classification make sense given the underlying pathologies of ALS. You'd assume that if age impacted the same white matter pathways, a validity test would be to show (i.e., quantify) that the same pathways predict age across data sets. But that's not what we really see here beyond just a qualitative comparison of the arcuate fasciculus. Why is that? How similar or dissimilar are the tractometry results across datasets for this analysis?

DISCUSSION: Age quintile bundle profiles are already in the supplement but we have added a paragraph on generalization to the Discussion section. These issues are to be expected given

what we know about the issues of data harmonization. Here, we are not combining across datasets (e.g., CamCan + HBN) in our analysis, and there are important differences between the datasets, beyond just data harmonization issues (e.g., age ranges in each study). In the HBN dataset, in which several different sites/scanners *are* combined, we apply a correction across sites, using NeuroCombat.

ACTION: We reran our HBN analyses using Neurocombat to harmonize across the two HBN sites (starting at line 460 in the methods). We added a discussion of validity and generalization to the Discussion section (starting at line 241).

- In the Methods it appears that the 3 data sets used in the age prediction differ substantially in their preprocessing routines. Is this a correct reading of the Methods? If so, how much could this variability explain the differences in model accuracies across data sets? Is there a way of understanding how specific preprocessing steps may impact model accuracy using this data?

DISCUSSION: CamCan and HBN were processed using almost identical pipelines (relying on qsiprep). The WH dataset was indeed processed using different methods. However, there are also other substantial differences between the datasets, so we don't think that the differences in preprocessing would explain all of the differences between performance in these datasets. One might imagine a study where preprocessing steps are varied within a single dataset to test this, but that is out of the scope of the current paper.

ACTION: We have elaborated on these differences more clearly when discussing generalization in the Discussion section (starting at line 241).

MINOR POINTS

- Fig 1. Define acronyms (e.g., CFA, CST) in the caption.

DISCUSSION: Thanks!

ACTION: We have added definitions of these bundle initialisms to the Figure 2 caption.

- Since the arcuate is a major predicting pathway in the age prediction, it makes sense to show/highlight it in Fig. 1 for contrastive comparison, in the same way that CST pathways are highlighted in Fig. 2.

DISCUSSION: Thank you.

ACTION: We have edited Figures 1, 2, and 3 to show both the arcuate and the CST.

- Fig. 2b is not mentioned in the Results.

DISCUSSION: Thank you!

ACTION: We have added a reference to Fig 2b to line 117 in the Results: "Figure 2b shows that SGL identified as potential disease biomarkers the diffusion measures in the CST from the cerebral peduncle to the corona radiata, agreeing with the previous study from which these data were extracted [20]."

- Is one of the reasons that the prediction is so good in the ALS case is that there are lesions in the descending CST pathways? These should be visible in the B0 images (or T2 FLAIRs if they are available). If so, this should be added to the text as interpreting these results will be different if it is picking up on subclinical pathologies in the white matter signal or if it is just detecting visible lesions.

DISCUSSION: We don't have access to these raw images anymore. We concede that it is possible that these results look so strong because they are based on image properties that would be visible in visual examination of patient scans.

ACTION: We have added to line 235 in the Discussion a sentence that acknowledges as much: "As with the original study, it is unclear whether our strong predictive performance based on image properties that would be visible in patient scans or whether it is identifying a new subclinical disease biomarker."

Reviewer #2:

The paper presents a method (Sparse Group Lasso, SGL) to analyse along-tract data derived from diffusion MRI. Along-tract profiling of various microstructural measures typically poses statistical problems due to the high dimensionality nature of the data. Here, data from 4 databases were employed to test the proposed SGL framework under two scenarios: classification and regression. In particular, the authors looked at the ROC AUC scores to classify ALS subjects based on their tract profiles. The authors found that SGL generally improved classification over a previously published approach based on Random Forest, while detecting the CST – a WM bundle typically affected by ALS - as an important feature. For regression, the authors used SGL to predict brain age on 3 different datasets.

The paper is well written and well-structured. As a recommendation, I believe that the reader would benefit from a summary of the input format for each dataset (i.e., it is unclear if the feature space was the same across datasets regarding the number of tracts and metrics.)

DISCUSSION: Thank you. Indeed, this was not explicitly stated.

ACTION: We added a sentence (line 453) stating that the feature space was the same for all datasets to the Methods section: "The resulting feature space was the same for all four datasets, with the FA and MD metrics at each of 100 nodes in eighteen bundles comprising 3600 features per subject." In the description of the pyAFQ pipeline, we also mention that the eighteen major tracts are enumerated in the Supporting Information (on line 353).

My major comment concerns the classification scenario. Although results may seem impressive, I wonder how difficult was the task at first glance. The authors failed to provide a comparison with standard classification approaches relying on more standard tract-averaging approaches. Would a simple tract average of FA disentangle both groups (e.g., using a standard deviation threshold)? I appreciate that the framework identified the CST as an important feature in an unsupervised fashion, but in this "straightforward" clinico-radiological experiment, one expects

the CST to be the driving feature for ALS. In other words, it is unclear if along tract-profiling provides added benefit for classification here (i.e., are 100 features per tract, per metric really necessary over 1 single feature per tract per metric?)

DISCUSSION: This is an important suggestion, because indeed, these approaches require more computational power and we need to make sure that they provide more accurate results. To do so, we added a comparison lasso linear model trained on bundle-averaged features, we call this bundle-mean lasso in the text. We find that the SGL-based approach outperforms the bundle-mean lasso in all of our pilot datasets. This replicates previous work on the along-tract profiling (PASTA) approach (e.g., Yeatman et al. 2012).

ACTION: We have added the bundle-mean lasso to the list of comparison models (lines 130 and 204 as well as the new Figure 4).

Minor comments:

“directional diffusion of water in each voxel [1].”

Stejskal, E. O., & Tanner, J. E. (1965). Spin diffusion measurements: spin echoes in the presence of a time-dependent field gradient. The journal of chemical physics, 42(1), 288-292.

ACTION: We have added the reference to Stejskal and Tanner to line 25.

This approach is exhaustive, but statistical power is compromised by a multiple comparison problem [7, 9, 10].

Chamberland, M., Raven, E. P., Genc, S., Duffy, K., Descoteaux, M., Parker, G. D., ... & Jones, D. K. (2019). Dimensionality reduction of diffusion MRI measures for improved tractometry of the human brain. NeuroImage, 200, 89-100.

DISCUSSION: This work is even more relevant now that we have incorporated PCA-based methods into the paper in response to reviewer #1’s comments.

ACTION: We have added this reference in the Introduction on line 41.

Fig. 1b) The red ROIs appear to be arbitrarily hand drawn over the corticospinal tract, which makes it confusing; e.g., were those starting-ending ROIs used to clip the CST profile or were they used for dissection only? I would say it is probably best to remove the drawing. The tract profile depicted in c) represents the traditional “bottom-to-top” CST FA profile with the well-known dip due to crossing fibers in the centrum semi-ovale. I would add the position along the tract along the x axis (c) so that uninitiated readers better appreciate the mapping from b to c.

DISCUSSION: Thank you! The ROIs were inclusion ROIs used to segment the CST bundle. But we agree that it was distracting and have removed the ROIs from the figure.

ACTION: As suggested, we removed the ROIs used for dissection. We also added the arcuate bundle here in response to reviewer #1's comment. Lastly, we have added an x label to the tract profile plots.

Material, dataset 2: "…fitting the diffusion tensor model [51] in every voxel using a robust fit [52]." Please specify that this was done on the b=1000 s/mm2 shell only.

DISCUSSION: Thank you.

ACTION: We have added this information to the Methods on line 384.

Typo: mahalnobis

DISCUSSION: Thank you!

ACTION: We have corrected this typo in the text on line 437.

Tractography: Please add details of missing parameters (step size, angular threshold, number of seeds, seeding method, number of streamlines per bundle per subject…)

ACTION: The tractography methods used for the WH and ALS datasets were different from those used for the HBN and Cam-CAN datasets. WH and ALS relied on deterministic tractography using the Matlab AFQ defaults for version 1.1 and 1.2, respectively. The HBN and Cam-CAN datasets relied on QSIrecon using MRtrix3 for multi-tissue CSD. We have added these tractography algorithm details to the Methods section in line 422.

Tractometry vs along tract profiling: I recommend paying special attention as to how both terms are mutually employed. In essence., Tractometry can refer to the quantitative mapping of multiples measures averaged over a set of tracts, as defined by Bells et al. 2011 and de Santis et al. 2014, whereas along-tract profiling specifically refers to the process of profiling the various MR-derived metrics, as defined by co-author J. Yeatman.

DISCUSSION: Thank you. This is a useful distinction.

ACTION: Throughout the manuscript, we now use the term "tract profiling" to refer to the along-tract profiling, and tractometry as the more general term, as suggested.

Overall, the framework provides a great addition to the existing AFQ software suite, and I find it worthy of publication at PLOS Computional Biology, conditional to the aforementioned comments.

Reviewer #3:

The authors present a multidimensional analysis method of white matter diffusion-based measures. It is based on sparse group lasso and allows to obtain informative features. It has several advantages, such as being able to perform analysis along the tracts, to automatically determine relevant features, and to perform classification or regression.

The method is interesting and very sound and can be very useful for neuroscientists.

The manuscript is well written and explains quite clearly the method, experiments, and results.

However, I think that more detail could be given on the results obtained. These should be better explained and analyzed. The figures contain considerable information but are not sufficiently explained in the text.

In addition, for example, I do not see displays of the tracts, where the different zones detected as relevant are highlighted (especially for the study of ALS). There are some small plots of the centroids, but these are very small and do not show the actual tracts. What is CFA?

ACTION: We now refer the reader both to the Supporting Information and a more explanatory text on pyAFQ for a description and depiction of these bundles (lines 122, 425, and 450). We have also defined the CFA initialism in the figure captions. Thank you!

Also, there is not enough comparison with previous methods.

DISCUSSION: That is correct. We now compare to LASSO PCR, and Elastic Net and also to tracometry using just the average, which we refer to as bundle-mean lasso.

ACTION: We added comparisons to the Lasso PCR, elastic net, and bundle-mean lasso for each dataset (see lines 129 and 203 as well as the new Figure 4).

On the other hand, the limitations of the method with respect to previous methods need to be better discussed, considering different aspects such as complexity, processing time, the minimum sample size required, selection of hyperparameters. Is it always better to use this method?

DISCUSSION: Thank you for this comment. One primary limitation is that the SGL approach takes more time. We have added a paragraph on execution time to the discussion section. Regarding minimum sample size required, the amount of data required is highly dependent on the strength of the signal in any given dataset so we don't feel comfortable making a general recommendation. However, SGL seems to adapt to the sample sizes in all four of our datasets (from ~50 to ~1600 subjects). And of course, in general, more data is better.

ACTION: We have added a paragraph on execution time to the Discussion section on line 269.

Also, how the transformation function is specified?

DISCUSSION: We mention the log transformation in the text, but the reviewer helpfully pointed out that we neglected to include how this is implemented in the code. Thanks.

ACTION: We have added technical details about this to the manuscript on lines 554 and 558, where we explain that we use scikit-learn's TransformedTargetRegressor with numpy's log and exp functions as the transform and inverse transform.

Finally, there is not enough reference to the supplementary material in the text (or I did not see it). A discussion of the most important results could be in the main manuscript

DISCUSSION: Thank you.

ACTION: We have added several additional pointers referring to the Supporting Information (lines 122, 127, and 425).
